# Intrameningioma Metastasis: Clinical Manifestation of Occult Primary Lung Carcinoma

**DOI:** 10.7759/cureus.704

**Published:** 2016-07-19

**Authors:** Muhammad Nadeem, Salman Assad, Humaira Nasir, Salman Mansoor, Innayatullah Khan, Hana Manzoor, Immad Kiani, Avais Raja, Touqeer Sulehria

**Affiliations:** 1 MBBS, FCPS, Department of Neurosurgery, Pakistan Institute of Medical Sciences, Islamabad, Pakistan; 2 Department of Neurology & Neurosurgery, Shifa Tameer-e-Millat University, Islamabad, Pakistan; 3 Department of Cardiology, Armed Forces Institute of Cardiology, Pakistan; 4 MBBS, FCPS Trainee, Department of Neurology, Shifa International Hospital, Islamabad, Pakistan; 5 MBBS, FRCS, Department of Neurosurgery, Shifa International Hospital, Islamabad, Pakistan; 6 Department of Neurology, Shifa International Hospital, Islamabad, Pakistan; 7 Department of Orthopaedic Surgery, Shifa College Of Medicine, Islamabad, Pakistan; 8 Radiology, Shaukat Khanum Memorial Cancer Hospital and Research Center

**Keywords:** meningioma, tumor, lung metastases

## Abstract

We report a case of lung carcinoma metastasizing into a meningioma in a 68-year-old female, who presented with progressively worsening right-sided hemiparesis and multiple episodes of adult onset epilepsy. Magnetic resonance imaging revealed an oval-shaped extra-axial hypointense lesion with a central hyperintense nodule in the left frontal region favoring a most probable diagnosis of a meningioma. Left frontoparietal craniotomy and excision of the tumor were carried out and histopathology with hematoxylin and eosin stain revealed a meningioma with metastatic adenocarcinoma and was confirmed by immunohistochemistry. The origin of metastasis was presumed to be from the lungs. A computed tomography (CT) scan of the chest with contrast showed a 3.1 x 2.9 cm mass with spiculated margins in the left lower lobe. Fine needle aspiration cytology (FNAC) proved it to be adenocarcinoma.

## Introduction

Though it is common for more than one tumor to occur in the same patient, metastasis from one tumor into another is very rare (tumor-to-tumor phenomenon). Metastasis can grow into a meningioma, neurilemmomas, and gliomas. It can even grow in the same location from where an intracranial meningioma has been removed [1]. We report a case of lung carcinoma metastasizing into a meningioma. Informed consent was obtained from the patient for this study.

## Case presentation

A 68-year-old female presented to our clinic with progressively worsening right-sided hemiparesis and multiple episodes of adult onset epilepsy. The patient had been previously seen by multiple specialty doctors for complaints of recurrent headaches, mood and behavior changes for the past six months. This was associated with decreased appetite and sleep. There was no history of head trauma, and no focal neurological deficits were found during the initial evaluations, general physical and neurological examinations. Four weeks ago the patient experienced a sudden onset of weakness in her right limbs. The patient developed an unsteady gait and her condition deteriorated to a point where she could neither walk nor stand without support. The patient experienced an episode of a severe headache with epilepsy. The episode, which subsided on its own, was witnessed by the patient’s relatives, who reported that the patient was jerking and had lost consciousness for two minutes. No episodes of tongue biting, rolling of eyes or loss of bowel or bladder control were witnessed. At the clinic, neurological examination of the patient revealed no cognitive deficits. Cranial nerve examination was normal. The power had significantly decreased in the right upper and lower extremities. Muscle tone was slightly decreased and reflexes were slightly weaker on the right side. The patient could not stand up or walk.

**Figure 1:**
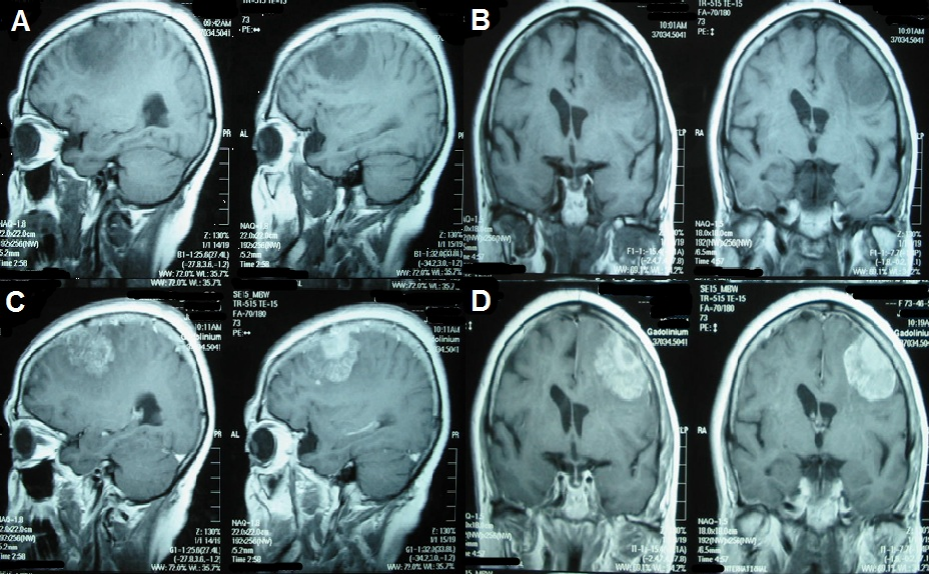
T1-weighted MRI Sagittal [A] and coronal [B], T1-weighted MRI demonstrate hypointense extra-axial lesion with a central hyperintense area. Post-contrast MRI sagittal [C], coronal [D] show enhancement of larger lesion with relatively more intense enhancement of central lesion.

**Figure 2:**
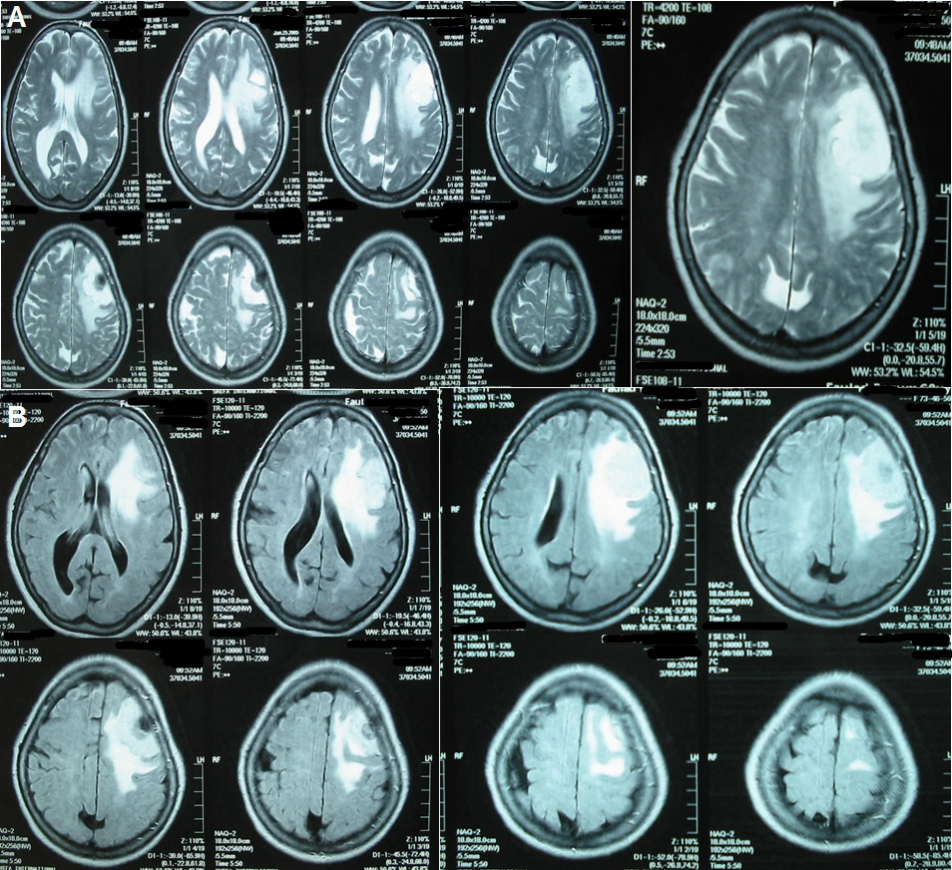
T2-weighted MRI gadolinium enhancement [A]. Axial T2-weighted MRI gadolinium enhancement shows a hyperintense lesion with the central hypointense area. The lesion is surrounded by perifocal edema. [B]. Fluid attenuation inversion recovery (FLAIR) sequence shows a hyperintense lesion with central hypointense area and edema.

**Figure 3:**
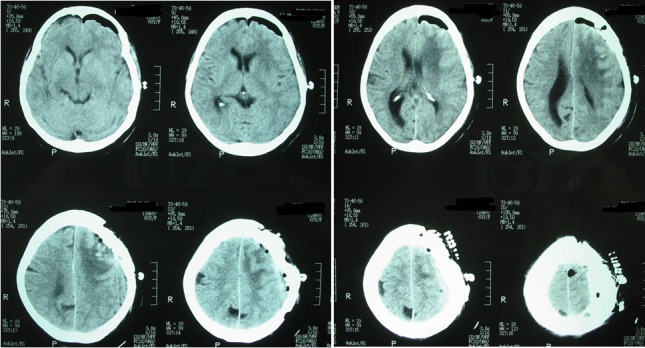
Postoperative brain CT scan Postoperative CT scan shows complete excision of the tumor with residual edema and pneumocephalus.

**Figure 4:**
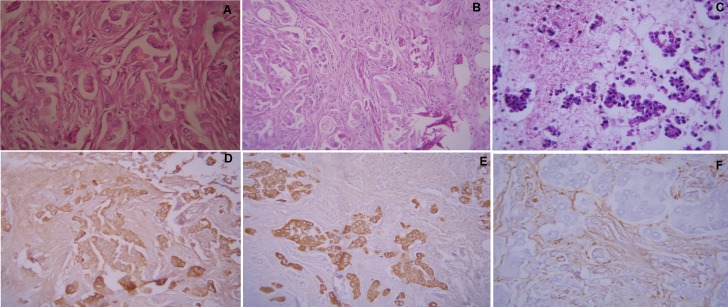
Histopathology and immunohistochemistry Histopathologic findings [A] Metastatic adenocarcinoma in a meningioma (H&E, 400×) [B, C] Cell block of FNAC of lung mass shows adenocarcinoma, pleomorphic cells with glandular formations (H&E, 400×). Immunohistochemistry findings [D] Epithelial membrane antigen (EMA) positivity in glandular formation as well as in the background meningioma cells. [E] Vimentin shows positivity in the meningioma cells, background adenocarcinoma is negative. [F] Pankeratin AE / AE3 shows strong positivity in a glandular component in the background negative meningioma cells.

**Figure 5:**
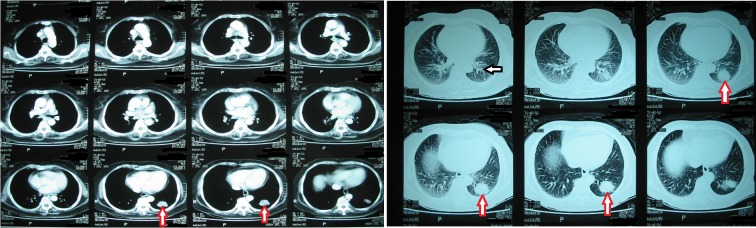
CT scan of chest A CT scan of the chest shows a 3.1 x 2.9 cm mass (red arrow) in the left lower lobe and mediastinal lymphadenopathy (black arrow).

T1-weighted magnetic resonance imaging (MRI) showed an oval-shaped extra-axial hypointense lesion with a central hyperintense nodule in the left frontal region (Figure 1). This nodule was hypointense on T2-weighted MRI with intense post contrast enhancement (Figure 2). The most probable diagnosis was a convexity meningioma. Her detailed systemic review was unremarkable. Left frontoparietal craniotomy and excision of the tumor were carried out. It had a dural base and was removed. Surgical resection of the tumor mass was carried out, and a postoperative CT scan demonstrated complete excision of the tumor with residual edema and pneumocephalus (Figure 3). Histopathology with hematoxylin and eosin (H&E) stain revealed a meningioma with metastatic adenocarcinoma and was confirmed by immunohistochemistry (Figure 4). The origin of metastasis was presumed to be from the lungs. A CT scan of the chest with contrast showed a 3.1 x 2.9 cm mass with spiculated margins in the left lower lobe, which was confirmed by FNAC as adenocarcinoma (Figure 5). A bone scan showed multiple metabolically active areas that indicated extensive metastasis. The patient survived only six months postoperatively despite radiotherapy and chemotherapy.

## Discussion

Intracranial meningioma is a relatively common tumor with an incidence of four per 100,000 and a female predominance of 2.5:1 [2]. A meningioma has been found to be the most common tumor to harbor metastasis although few cases of metastasis into neurilemomas and gliomas have also been reported. Various predisposing factors have been suggested like increased vascularity, low metabolic rate, high collagen and lipid content, cellular signaling, poor immune surveillance of tumor tissues, loss of tumor suppressor gene, and complex interactions involving hormonal factors. However, the precise mechanism of this rare occurrence remains unidentified [2]. Moody, et al. suggested that psammoma bodies (PBs) confer protection against metastatic deposits and these bodies were absent in this case [3]. Interestingly, in the case of metastasis to intracranial lesions, metastases to the remainder of the brain are rare.

Hyo Sung Han, et al. suggested a criterion to assess a true tumor-to-tumor metastasis as follows. Firstly, the metastatic focus should be at least partially enclosed by a rim of tumor tissue, and the existence of metastasizing primary tumor must be proven and compatible with metastasis [4]. The present case fulfilled the above-mentioned criteria. Approximately 90% of metastases arose from breast and lung carcinomas. Cases from kidney, prostate, thyroid, endometrial, gall bladder, esophagus, lymphoma, malignant melanoma, and colon have also been reported. A review of the literature by Han, et al. revealed that cases detected by autopsy were twice as prevalent than surgical cases, and the metastasizing tumor was always found to be widely disseminated [4].In this case also, the tumor was widely disseminated. The majority of meningiomas showed uniform enhancement on CT or MRI studies, but many meningiomas showed non-enhancing areas caused by cystic or necrotic change, hemorrhage, and calcification. Meningiomas present with variable radiological enhancements ranging from non-homogeneous to homogeneous. Sayegh, et al. documented a meningioma case with homogeneous enhancement [5]. Metastasis might exhibit more intense enhancement than the background benign lesion. Radiotherapy is usually suggested for the optimal management of the tumor bed. Though radiotherapy and chemotherapy were given to the patient postoperatively, the patient survived only six months after surgery due to widespread metastases.

## Conclusions

A patient with a meningioma showing areas of abnormal enhancement can have widespread metastatic disease even if a primary is unknown. Tumor-to-tumor metastasis is a rare but important phenomenon, particularly in the case of invasive tumors. Transportation of malignant cells from the lungs can be easily explained by the extensive and high blood flow in the meningioma vessels. The only diagnostic approach towards identifying an intrameningioma metastatic lesion is through biopsy of the tumor. An optimal management approach towards treatment of superimposed malignancies needs to be developed.
